# Comprehensive Roles and Future Perspectives of Exosomes in Peritoneal Metastasis of Gastric Cancer

**DOI:** 10.3389/fonc.2021.684871

**Published:** 2021-06-29

**Authors:** Xiangliu Chen, Haiyong Wang, Yingying Huang, Yanyan Chen, Chuanzhi Chen, Wei Zhuo, Lisong Teng

**Affiliations:** ^1^ Department of Surgical Oncology, The First Affiliated Hospital, School of Medicine, Zhejiang University, Hangzhou, China; ^2^ Department of Cell Biology, School of Medicine, Zhejiang University, Hangzhou, China

**Keywords:** gastric cancer, exosomes, peritoneal metastasis, microenvironment, pre-metastatic niche

## Abstract

Gastric cancer (GC) is one of the most prevalent digestive malignancies. A great number of patients at first visit or post curative resections are diagnosed with widespread metastasis within the peritoneal cavity. Overwhelming evidence has demonstrated that exosomes, a variety of biologically functional extracellular vesicles comprising active factors, mediate the progression and metastasis of GC. Although the regulatory mechanisms of exosomes remain fairly elusive, they are responsible for intercellular communication between tumor cells and normal stroma, cancer-related fibroblasts, immune cells within the primary tumor and metastatic niche. In this review, we provide new insight into the molecular signatures of GC-associated exosomes in reprogramming the tumor microenvironment and the subsequent promotion of peritoneal metastasis—including infiltration of the gastric wall, implantation of tumor cells onto the pre-metastatic peritoneum, and remodeling of the pre-metastatic niche. Based on this review, we hope to draw a more general conclusion for the functions of exosomes in the progression and peritoneal metastasis of GC and highlight the future perspective on strategies targeting exosomes in prognostic biomarkers and therapy for peritoneal metastasis.

## Introduction

Gastric cancer (GC) is the fifth most lethal malignancies worldwide and the third dominating cause of cancer-related death, responsible for 7% of cancer cases and 9% of the deaths (1), especially in East Asia, such as Japan, Korea, and China (2). Generally, peritoneal dissemination (PD) is the most common distant metastasis mode for GC and the most important factor leading to shortened survival of patients (3). Without treatment, a 5-year survival rate of GC patients with peritoneal metastases (PM) is 2% with the median survival time of 3–5 months (4). Despite radical operation of gastric cancer, around 50% of GC patients with advanced disease develop peritoneal metastasis (5). Nowadays, the combinations of platinum agents with addition of taxane, fluoproyrimidine, or anthracyclines are usually regarded as the standard regimens for advanced or recurrent GC, including peritoneal metastases, but the efficacy of these systemic chemotherapy drugs for patients with PD is still limited (6).

Exosomes are membrane vesicles generated from the multivesicular endosomes with size ranging from 30 to 100 nm in diameter (7). Exosomes are released by all kinds of malignant and normal cells and are distributed in various bodily fluids such as plasma, urine, saliva, and malignant effusions (8). Exosomes display significant roles in intercellular communications to local and remote cells and organs, by selectively transferring its cargo (protein, lipids, DNA, RNA, and membrane) (9, 10). Notably, extensive studies have validated that exosomes contribute to different aspects of GC progression and PM by promoting primary tumor cells growth, invasion, and remodeling the peritoneal microenvironment to make it suitable for metastatic niches (11, 12). Exosomes participate in pluripotent cell functions in PM, inducing cancer-associated fibroblasts (CAFs), epithelial–mesenchymal transition (EMT) of cancer cells, mesothelial-to-mesenchymal transition (MMT) of peritoneal cells, angiogenesis, and immune suppression, changing the environment in both local and pre-metastatic stroma (13, 14).

**Figure 1:**
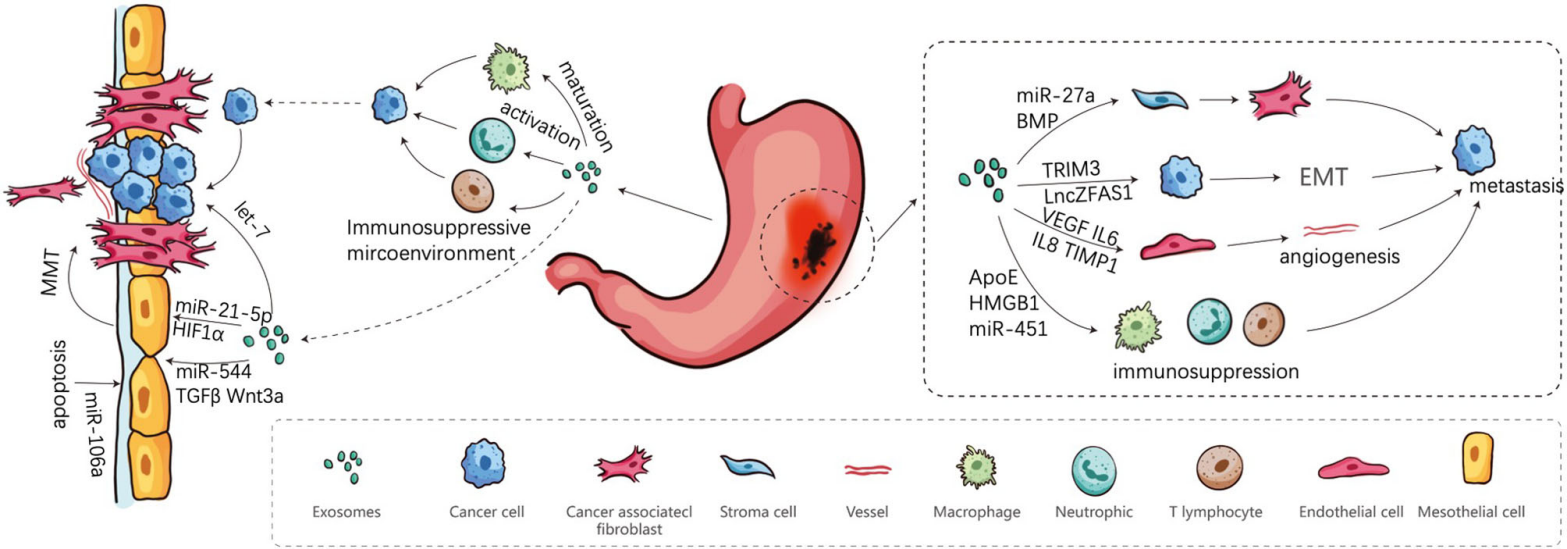
Involvement of exosomes in peritoneal metastasis Cascade. Firstly, cancer cells are shed from the primary sites, and secondly transported in the peritoneal cavity. Finally, cancer cells adhere to the MCs and implant in peritoneum.

In this review, it is stated that exosomes secreted by GC cells and other stroma cells have internal and external effects on GC growth, invasion, and PM, as summarized in **Figure 1** and **Table 1**. We have systematically clarified the roles of exosomes in all conceivable steps of PM, in which cancer cells spread from primary to peritoneum and finally form metastatic lesions. Since the characteristics of exosomes are dependent on their donor cells and the conditions of their formation, clarifying complete property practices of exosomes is conducive to their applications. The most thorough understanding of the content of the exosomes and underlying mechanisms on PM will be promising in aiding the novel discovery of potential diagnostic molecules and targets for treatment of GC.

**Table 1:** Function of exosomes in progression and peritoneal metastasis.

Exosomal cargo	Donor cells	Recipient cells	Role/Mechanism	Ref.
miR-27a	Gastric cancer	Stroma fibroblasts	Tumorigenesis and metastasis/CSRP2	(15)
BMP	Gastric cancer	Pericytes	Metastasis/PI3K and MEK pathway	(16)
TGF-β	Gastric cancer	MSCs	Metastasis/Smad	(17)
TRIM3/ ZFAS1	Gastric cancer	Gastric cancer	Growth and metastasis/EMT	(18, 19)
circNRIP1	Serum	Gastric cancer	metastasis/miR-149-5p-AKT-mTOR	(20)
\	Gastric cancer	PMCs	MMT/ERK pathway	(21)
miR-21-5p	Gastric cancer	PMCs	MMT/TGF-β pathway	(22)
miR-106a	Gastric cancer	PMCs	Apoptosis/SMAD7	(23)
Wnt3a	Gastric cancer	PMCs	Tumor invasion	(24)
VEGF,TIMP-1,IL6,IL8,FGF	Gastric cancer	Endothelial cell	Angiogenesis	(25, 26)
miR-130a	Gastric cancer	Endothelial cell	Angiogenesis/c-MYB	(27)
miR-135b/155	Gastric cancer	Endothelial cell	Angiogenesis/FOXO1/3	(28, 29)
MET	Gastric cancer	macrophage	Proinflammatory environment/ NF-κB /IL-1β	(30, 31)
\	Gastric cancer	PD1+TAMs	Immunosuppression/IL10	(32)
\	Gastric cancer	MSCs	Inflammatory environment/ NF-κB	(33)
miR-107	Gastric cancer	MDSCs	Immunosuppression/DICER1,PTEN	(34)
miR-451	Gastric cancer	Th17	Angiogenesis/mTOR	(35)
HMGB1	Gastric cancer	neutrophils	Migration/ NF-κB	(36)
\	Gastric cancer	CD8+Tcell	Immunosuppression/cytokine	(37)
\	CAF	Gastric cancer	Invasion/MMP2	(38)
\	Gastric cancer	PMCs	Adhesion/fibronectin,laminin gamma10	(39)
CD44H,CD44v6	Gastric cancer	Gastric cancer	Growth,angiogenesis	(40)
Let-7 miRNA	AZ-P7a	Gastric cancer	,Tumorigenesisi, PM	(41)
TGF-β,Wnt3,HIFα,Src	Gastric cancer	Stromal cell	MMT	(42)
miR-139	CAF	Gastric cancer	Growth and metastasis/MMP11	(43)
ApoE	TAMs	Gastric cancer	Migration/PI3K-AKT	(44)
IL6,IL8,ICAM1,GRO	Omentum	Gastric cancer	Progression/metastasis	(45–48)
miR-544-5p	PLF	PMCs	MMT/PLZF	(49)
\	MA	PMCs/GC	Metastasis/ MMT, EMT	(50, 51)

## Tumor-Derived Exosomes Assist Gastric Tumor Cells in Detaching From the Primary Tumor Sites

The PM cascade of GC is composed of a series of sequential events that GC cells have to accomplish, including metastatic spread of primary tumor mass, formation of pre-metastatic niches and final planting of metastatic sites (13). These exosomes, either tumor-derived exosomes or stromal cell-derived exosomes, are mobile elements participating in the first process—the spread of GC cells out of serosa, recruitment and activation of fibroblasts, induction of angiogenesis, and EMT promotion.

### Tumor-Derived Exosomes Are Involved in Converting Stroma Cells to CAFs

Cancer-associated fibroblasts (CAFs) are the major part of cellular constituents of the cancer stroma and are indicative of the myofibroblast phenotype and strong contractility, which result in the remodeling and hardening of the extracellular matrix (ECM) and provide an appropriate microenvironment for cancer cell mobility and metastasis (52). Apart from the soluble factors, exosomes play an essential role in connection between cancer cells and CAFs. In a study, researchers found an extremely high level of miR-27a in exosomes secreted from patients’ sera and cell lines of GC, which may contribute to the transition of stroma fibroblasts into CAFs by targeting a downstream target cysteine and glycine-rich protein 2 (CSRP2). Similarly, over-expression of miR-27a CAFs could increase the proliferation, motility, and invasion abilities of GC cells *in-vitro* and *in-vivo* (15). This indicates that GC-derived exosomes alone are competent in the induction of functional changes from normal fibroblasts to pathogenic CAFs. Similar studies have verified the potential of GC exosomes in converting normal stroma to CAFs. Stroma cells such as normal pericytes and mesenchymal stem cells could be induced into CAFs by GC exosome-mediated BMP transfer (PI3K/AKT and MEK/ERK pathway) and TGF-*β* transfer (TGF-*β*/Smad pathway) respectively (16, 17).

As of now, these studies have confirmed that tumor-derived exosome are able to induce the change from normal stroma to cancerous stroma, which is essential for cancer progression of cancer cells and secondary metastatic growth. Exosomes derived from GC could deliver functional elements to induce CAF formation by activating AKT, ERK, and TGF-*β*/Smad signaling pathway, which are consistent with the studies of other cancers (53, 54).

### Tumor-Derived Exosomes Induce EMT Process of GC

EMT allows epithelial cancer cells to lose epithelial-like traits and acquire a mesenchymal phenotype of the migratory and invasive characteristics for morphogenesis, which is an essential part of GC dissemination and metastasis (55, 56). Tumor-derived exosomes carry proteins and non-coding RNAs (lncRNA, miRNA, and circRNA) that directly enhance the invasive and migratory capabilities of GC cell (57). For example, exosomal Tripartite motif-containing 3 (TRIM3) and long non-coding RNA ZFAS1 of GC cells can promote growth and metastasis of cancer cells through mediating stem factors and EMT markers *in-vitro* and *in-vivo* (18, 19). Zhang et al. detected higher circNRIP1expression in GC tissues *via* RNA-seq analysis and transmission of circNRIP1 by exosomal communication between GC cells relative to normal tissues. Additionally, circNRIP1 is able to thicken the proliferation, migration, invasion and the expression of EMT markers in GC cells *via* sponging of the miR-149-5p/AKT/mTOR pathway. Formidably, exosomal circNRIP1 functions as a promoter of peritoneal metastasis in BABL/c nude mice (20). To some extent, exosomes derived from GC cells can transmit functional elements participating in the process of EMT on themselves, which is inevitable for onset of metastasis.

## Roles of Exosomes in Pre-Metastatic Niche Formation of Gastric Cancer

Post exfoliation from the serosa of the stomach into the abdominal cavity, GC cells survive by creating a favorable microenvironment for subsequent metastases in secondary organs. The “seeds and soil” hypothesis has been instrumental to our understanding of PM, which depends on the characteristics of cancer cells (seeds) and target organs (soil) (58). For example, pancreatic ductal adenocarcinoma patients who developed liver metastasis produce tumor-derived exosomes carrying high macrophage migration inhibitory factor (MIF), which could induce liver pre-metastatic niche formation and subsequently enlarge liver metastatic burden (59). In the progression of PM, exosomes play multifaceted roles in affecting pre-metastatic microenvironment through inducing mesothelial-to-mesenchymal transition (MMT) of peritoneal mesothelial cells (PMCs), supporting the pro-angiogenic function of endothelial cells and immune evasion of tumor cells (57).

### Tumor-Derived Exosomes Contribute to Breach the Barriers for Tumor Invasion Within the Pre-Metastatic Niche

Intraperitoneal metastasis of GC is formed by reciprocal interaction between exfoliated cancer cells and peritoneal cells, especially PMCs (60). In particular, MMT of PMCs could present the peritoneum more willing for cancer cells’ attachment and invasion and contributes to pre-metastatic niche forming by promoting its vascularization (61). Accumulated studies demonstrate that tumor-associated exosomes can disrupt mesothelial barrier to facilitate peritoneal metastasis.

Deng et al. found that exosomes derived from GC cell lines could damage the mesothelial barrier and elicit PMCs to undergo MMT and effect apoptosis *in-vivo* and *in-vitro*. They also observed that at the molecular mechanism, ERK pathway plays a crucial part in MMT of PMCs, but not apoptosis. Also evident is the exosomal mediated increased expression of MMT-related protein, including Vimentin, Fibronectin, and Collagen-I through ERK1/2 activation (21). However, the element in exosomes responsible for the MMT of PMCs remains to be fully identified and explained. Recently, a study revealed that this process could be induced by GC-derived exosomal miR-21-5p targeting SMAD7 to activate TGF-*β*/smad pathway and to effect the increase of invasion and attachment of PMCs (22). Subsequently, Zhu at al. also identified that GC-derived exosomes enriching miR-106a destroy the mesothelial barrier by breaking the PMCs’ balance of apoptosis and proliferation through targeting SMAD7 (23).

The progression of peritoneal metastasis of GC is dependent on the breaching of barriers of the peritoneum. Intriguingly, PMCs undergoing MMT process acquire high migratory and invasive abilities which help them invade the submesothelial space and induce formation of fibrosis and angiogenesis; so it is a crucial component of premetastatic niche (62). In this regard, there is still a diverting study as to the exact function of PMCs during the first phase of primary cancer progression. Tanaka et al. detected that PMCs could cover the gastric wall and infiltrate into submucosal to create a suitable niche for tumor invasion. Meanwhile, GC cells also release exosomes involving Wnt3a, thus promoting PMC infiltration. PMCs in turn boost cancer cells’ invasion out of the stomach and the formation of peritoneal metastatic lesions (24). Tumor-associated exosomes would facilitate the initiation of the invasion-metastasis cascades by taking part in the clearance of physiological peritoneum barriers which restrain metastasis of tumor cells.

### Tumor-Derived Exosomes Participate in Angiogenesis

To ensure adequate nutrition and oxygen supply, endothelial cells begin neoangiogenesis in the primary tumor mass and pre-metastatic microenvironment. GC cells and endothelial cells can interact with each other through exosomes, which can partially induce the angiogenesis of the later to regulate the peritoneal metastasis of GC (63). Tumor cells release exosomes loaded with pro-angiogenic factors such as VEGF, TIMP-1, IL-6, IL-8, and FGF and carry paracrine signaling elements and miRNAs to drive genetic expression towards angiogenesis (25, 26). MiRNAs (miR-130a, miR-135b, miR-155) wrapped in tumor-derived exosomes have been found to promote GC angiogenesis and metastasis through c-MYB, forkhead box O1 (FOXO1), and FOXO3 respectively (27–29). Notably, these miRNAs have promotional effect on viability and migration of human umbilical vein endothelial cells. Tumor-derived exosomes, as well as exosomes secreted from stromal cells, release factors to recruit and activate endothelial progenitor cells to enhance angiogenic response (25). In pre-metastatic niche, exosomes released by normal stromal cells have a role in microenvironment homeostasis, but tumor-derived exosomes are actively involved in the formation of pre-metastatic niche. Nevertheless, we still do not know whether exosomes derived from other several tumor-associated stromal cells play the same role in PM of GC as other types of cancers.

### Tumor-Derived Exosomes Mediate Immunosuppression Within the Pre-Metastatic Niche

It is important for tumor cells to escape immunological elimination and establish an immunosuppressive microenvironment in the primary tumor sites and pre-metastatic niche (64). GC-derived exosomes exist in neoplastic lesions or biologic fluids of cancer patients possessing immunosuppressive molecules that mediate immune cell dysfunction and transform the suitable microenvironment for isolated tumor cells survival and metastasis, including suppression for immune cell activation and induction of immunosuppressing cells (65). Wu et al. found that exosomes derived from GC can stimulate the activation of NF-*κ*B pathway in macrophages to promote GC progression through regulating proinflammatory microenvironment (30). However, the question of which substance from exosomes promotes the activation of macrophages has not been analyzed clearly. Recently, exosomal mesenchymal–epithelial transition factor (MET) was found to be higher in *H. pylori*-infected GC cells and educated the macrophages toward pro-tumorigenesis *in-vivo* and *in-vitro* (31). GC-derived exosomes could effectively elicit monocytes to differentiate into PD1+ tumor-associated macrophages (TAMs) with M2 surface profile and functional characteristics. These cells then secrete a great number of IL-10 to destroy the antitumor cells (CD8+ T cells) and thereby create a favorable immune microenvironment for GC angiogenesis and metastasis (32). Furthermore, mesenchymal stem cells (MSCs) are an important component of tumor immunomodulation. Shen et al. revealed that GC-derived exosomes affect the biological functions of MSCs through NF-*κ*B pathway to activate immune cells, maintain inflammatory conditions, and stimulate tumor metastasis (33). For example, MSCs can facilitate macrophage phagocytosis and the activation of CD4/CD69 (the early activation of T cell markers) double-positive T cells. In addition, exosomes transferring miRNA-107 from tumor expand and activate myeloid-derived suppression cells to promote immunosuppression in the tumor microenvironment and help GC cells’ growth and survival by targeting DICER1 and PTEN gene (34). Exosomes which comprise of miR-451 from GC targets mTOR to enhance differentiation of T-helper 17 (Th17) cells which infiltrate into GC sites to promote angiogenesis and support cancer progression (35). Exosome-mediated transfer of high mobility group box-1 (HMGB1) from GC cells activates neutrophils by NF-*κ*B pathway, which promotes tumor cell’s migration (36). Recently, a study demonstrated that exosomes derived from GC can regulate CD8+ T cell gene expression and cytokine secretion patterns to establish an immunosuppressive microenvironment for metastatic niche formation (37).

In brief, these findings point out that exosomes play an important role in remodeling a robust pre-metastatic niche, which allows for immune evasion of tumor cells, especially GC-derived exosomes. Those exosomes delivering to immune cells can educate macrophages and neutrophils into protumor phenotype, impair CD8+ T cells’ activation, induce MSCs for tumor immunomodulation, and increase Th17 differentiation within primary tumor sites and pre-metastatic niche. With a better understanding of the roles of exosomes in immune regulation, it may provide novel and efficient antitumor therapies in PM of GC.

## Exosome-Educated Cells in the Pre-Metastatic Niche Promote Metastasis

GC-derived exosomes could facilitate exfoliative tumor cells’ invasion, settlement, colonization, and metastasis within the pre-metastatic mass while inhibiting the host anti-tumor immune response. Following the successful formation of the pre-metastatic microenvironment, activated PMCs, CAFs and TAMs can then support peritoneal implantation of GC through their own exosome secretion (24, 32, 38). Experimental evidence has shown that PMCs internalizing GC-derived exosomes could up-regulate expression of adhesion molecules (fibronectin and laminin gamma 1) and significantly promote adhesion with exfoliated GC cells, while the same is not true for internalization of mesothelial cell-derived exosomes (39). Cluster of differentiation 44 (CD44), an adhesive molecule, is important for attachment of tumor cells to the peritoneum (66). Simultaneously, CD44 also serves a similar role in attachment between tumor cells and exosomes. Malgorzata et al. discovered that exosomes isolated from GC cell lines expressed CD44H and CD44v6 involved in interaction with GC cells to support GC growth and angiogenesis (40). The study manifests that exosomes act as macro-messengers in delivering molecular cargo and signals to enhance their own carcinogenicity *via* autocrine tumor loops. In another study, researchers disclosed that AZ-P7a cells, a high peritoneal-metastatic potential gastric cancer line, secrete exosomes containing abundant let-7 miRNA family as compared to other metastatic cancer lines. And let-7 miRNAs were released into the extracellular matrix to maintain their tumorigenesis and PM (41). This study indicated that exosomes secreted by secondary metastatic lesions rather than primary cancer mass are more inclined to promote proliferation and invasion. Recently, TAMs play a distinctly supportive function to promote GC cells’ invasion, in which TAMs transfer tumor-derived extracellular vesicles containing RNA and proteins (TGF-*β*, activated Src, Wnt3, and HIF1*α*) to peritoneal stromal cells and elicit a pro-tumor microenvironment, such as induction of MMT of PMCs (42).

Overall, previous data demonstrate that tumor-derived exosomes may not only assist tumor cells’ attachment to mesothelial cells, but also further facilitate tumor metastasis and progression. There are many factors that promote PM occurrence of GC, but the role of tumor-derived exosomes in this process remains to be further investigated. Additionally, exosomes carry various known and unknown factors which may play functional and non-functional roles in those recipient cells, so the characteristics and contents of exosomes should be continuously clarified.

## Roles of Exosomes Derived From Other Sources in the Processes of PM

The occurrence and development of PM induced by exosomes are complex and dynamic. Not only tumor derived exosomes are actively involved in PM of GC as described above, non-tumor derived exosomes with potential pro-oncogenic functions are also involved in promoting tumor metastases.

### Exosomes Secreted by Tumor-Reprogrammed Normal Cells Play Supporting Roles in PM

CAFs induced by cancer cells can secrete functional exosomes to accelerate cancer progression and metastases. Recent evidence showed that CD9-positive exosomes from CAFs may promote the migration and invasive ability of cancer cells through MMP2 activation in scirrhous-type gastric cancer (38). Additionally, CAF-derived exosomes can also deliver miR-139 to suppress the progression and metastasis of GC by down-regulating MMP11, which can facilitate cancer cells’ migration (43). Aside from this effect, CAFs also increase basement membrane permeability for cancer cells’ invasion by stretching and pulling it to seal gaps (67). As for exosomes derived from immune cells, TAMs with M2 phenotype transfer apolipoprotein E (ApoE) by exosomes to trigger the activation of PI3K–Akt signaling pathway in GC cells and subsequently aggregate tumor cells’ migration (44). In addition, one such study demonstrated that omental tissue-derived exosomes of gastric cancer patients shuttle higher levels of interleukin-6 (IL-6), interleukin-8 (IL-8), intercellular adhesion molecule-1 (ICAM-1), growth related oncogene (GRO), basic fibroblast growth factor (bFGF), adiponectin, and C-C Motif Chemokine Ligand (CCL4) (45), which had been studied as GC-related cytokines facilitating gastric cancer proliferation, angiogenesis, invasion, immunomodulation, and peritoneal metastasis (46–48). These studies demonstrated that tumor-derived exosomes benefit from the production of tumor-reprogrammed stromal cells, which simultaneously secrete functional non-tumor-derived exosomes capable of promoting cancer progression and metastases.

### Roles of MA-Derived Exosomes in PM

The abdominal cavity is a huge physiological cavity, and peritoneal fluid is rich in various functional factors. The GC patients’ peritoneal fluids could contain numerous exosomes derived from tumor cells and tumor-associated cells, with capabilities to boost the development of PM. Exosomes derived from peritoneal fluid of GC patients with PM carried higher miR-544-5p, suppressing the promyelocytic leukemia zinc finger (PLZF) expression in peritoneum cells, in which suppression induces MMT of peritoneum and results in increased invasion potential of abdominal free tumor cells (49). Similarly, gastric malignant ascites (MAs)-derived exosomes also participate in the development of peritoneal metastasis. Experimental evidence has shown that MA-derived exosomes enhance the level of fibroblast activation protein (FAP), alpha-smooth muscle actin (*α*-SMA), and fibronectin, which are CAF specific markers. Functionally, PMCs cause MMT by exosomal effected increase of the capacities of proliferation and formation of peritoneum fibrosis through TGF-*β*1 *in-vitro* and *in-vivo* mouse models (50). Moreover, Hu et al. demonstrated that MA-derived exosomes facilitate GC cells’ invasion by the up-regulation of EMT signaling and peritoneal metastasis in GC cells’ intraperitoneal metastatic xenograft mouse model (51). Therefore, exosomes secreted from various tumor-associated cells can participate in the formation of pro-oncogenic abdominal microenvironment and contribute to the progression of PM in GC patients. The peritoneal fluid-derived exosomes of GC patients are different in molecular, genetic, and functional heterogeneity, so it is necessary to further explore the division of labor of different cells derived exosomes in these stages of PM.

## Exosomes as Pre-Metastatic Niche Biomarkers and Therapeutic Applications

Exosomes possess several apparent advantages as biomarkers of predicting the occurrence and development of PM, as they are exceedingly stable, ample, and tumor-specific. Therefore, they are promising biomarkers within the blood and ascites that deserve abundant investigation in this deadly cancer. In addition, exosomes are extremely potent intercellular communicators and are expected to bring great breakthrough in therapies for various diseases, including PM of GC.

### Exosomal miRNAs as Biomarkers of PM

Given that exosomes are widely present in various body fluids (blood, urine, ascites) and contain many inclusions (DNA, RNA, protein), exosomes are suggested to be optimal candidates for relatively non-invasive method of diagnosis of diseases and evaluation of therapy efficacy (68). Increasing pieces of evidence have shown that exosomes have a great potential to act as biomarkers for PM of GC. Thus, highly sensitive and specific molecular markers are necessary to predict and assess tumor burden in the peritoneal cavity. Furthermore, liquid biopsy is a common strategy, in which circulating tumor cells and cell-shed exosomes could be detected from body fluids, including ascites and plasma (69). Fortunately, a team performed a high throughput sequencing of ascite-derived exosomes among eight paired GC patients with peritoneal dissemination before and after intraperitoneal chemotherapy, of which three had non-malignant disease. They detected an increase in MA-derived exosomes associated with five miRNAs (miR-760, miR-6821-5p, miR-4745-5p, miR-200a-5p, miR-4741, and miR-320) in the malignant disease group as compared to those individuals with non-malignant diseases (51). Interestingly, some identified miRNAs in exosomes could be down-regulated or up-regulated in those GC patients who were after their peritoneal chemotherapy. Tokihisa et al. analyzed the miRNA microarrays among six MA samples and 24 peritoneal lavage fluid (PLF) samples and demonstrated that exosomal miR-21 and miR-1225-5p were significantly up-regulated in those MA and PLF of serosa-invasive GC and associated with serosal invasion. What is more, the two candidate miRNAs may potentially act as biomarkers of peritoneal recurrence following curative GC resection (70). More recently, there was a miRNA expression profile analysis of peritoneal lavage fluid derived exosomes among patients with PM and patients without PM (71). They identified that the expression of miR-21-5p, miR-92a-3p, miR-233-3p, and miR-342-3p was significantly higher in former samples and positively associated with pathological serosal exposure and the extent of peritoneal cancer throughout the peritoneal cavity. In contrast, exosomal miR-29 family was down-regulated in PM (+) patients, and the expression of miR-29b-3 was negatively correlated with worse peritoneal recurrence-free survival and overall survival. This result shows that the role of miR-21 is similar to previous ones. Similarly, Ohzawa et al. revealed that down-regulated miR-29s are a strong risk factor of peritoneal recurrence and worse overall survival. This indicates that the expression of miR-29s in MA or PLF could be a reliable biomarker to assess the probability of peritoneal recurrence in patients undergoing curative surgery (72).

Thus, the clinical utility of liquid biopsy in the detection of exosomal biomarkers can potentially be an important diagnostic and prognostic tool for the assessment of PM in GC (69). Owing to individual differences, the tremendous heterogeneity exists in exosomes of GC patients, and it remains unknown whether exosomal biomarkers from body fluids present adequate sensitivity and specificity to assess the authentic situation of PM’s occurrence and recurrence. This requires sufficient samples of GC patients and multi-center research to screen for more sensitive and specific exosomal markers.

### Exosomes as Therapeutic Applications Targeting PM of GC

As yet, systemic chemotherapy is considered the standard treatment for PM of gastric cancer, but the survival outcomes of patients are still poor (73). Nevertheless, effective methods for treatment of PM are extremely needed. At present, therapeutic exosome-based strategies are an emerging treatment and currently focus on exploration of the targeting moieties of exosomes to primary cancer cells and metastatic tumor (74). Exosomes could become a new avenue for transporting anti-cancer molecules and drugs in the treatment of cancer because of their low immunogenicity, high biocompatibility, high efficacy of delivery and biodegradable characteristic (75). One of the first demonstrations for this potent capability of exosomes came from a study in which exosomes were engineered to target the recipient cells with high Her2 expression by displaying anti-Her2 single chain variable fragment on the exosome surface (76). Similarly, modified exosomes loaded with anticancer agent paclitaxel and doxorubicin specifically interact with *α*v integrin-positive breast cancer and pulmonary metastases with overexpressed sigma receptor respectively, exhibiting high anticancer efficacy (77, 78). One research group synthesized engineered exosome-thermosensitive liposomes hybrid NPs, which efficiently penetrated into peritoneal metastasis nodules and released payloads to inhibit tumor development in cancer line-derived xenografts and patient-derived tumor xenografts (79). The study provides evidence to support the feasibility of therapeutic exosome-based approach in improving drug delivery and treatment of metastatic peritoneal carcinoma.

For instance, exosome-mediated delivery of miRNA (miR-374a-5p, miR-214) inhibition could possibly antagonize the effect on growth, migration, and chemoresistance of GC cells to retrieve drug effectiveness (80, 81). Zhang et al. revealed that hepatocyte growth factor (HGF) siRNA packed in exosomes could inhibit proliferation and migration of cancer cells and vascular cells through activation of MAPK, PIK3, and Stat3, as well as up-regulating VEGF expression (82). To date, there are few studies that have focused on exosomes for targeting peritoneal metastasis of GC. TRIM3-overexpression of exosomes can decrease the size and number of metastatic tumor nodes in PM models (82). Additionally, silencing of exosomal miR-21-5p could block MMT of peritoneum through attenuating TGF*β*/SMAD pathway (22). This targeted technology has potential for suppression of metastatic niche progression and is an efficient area of future chemotherapeutic study.

Tumor-derived exosomes preparing the pre-metastatic niche must undergo homing to remote organs and other sites. Exosome proteomics have demonstrated that specific expression patterns of integrin largely contribute to this process. Exosomal integrins *α*6*β*4 and *α*6*β*1 were related with lung metastasis, and exosomal integrin *αvβ*5 was associated with liver metastasis. Nevertheless, targeting these integrins could restrain exosomes’ uptake, as well as respective metastasis (83). The study serves as an inspiring proof of the concept that using specific exosomal integrin blockers can efficiently battle metastasis. Various malignancies exhibit unique exosome profiles and have distinct propensity to metastasize to specific sites (84). Additionally, there was a direct proof verifying that tumor-derived exosomes transmit signals over long ranges to metastatic niches *in-vivo* (85). Therefore, further elucidating the homing pattern of tumor-derived exosomes which bind to peritoneal cells could surface information on novel tumor biomarkers and is a promising therapeutic strategy in avoiding the occurrence of PM. Given the immaturity of exosome biology and strenuous preliminary work of clinical transformation, more effort is needed in the attempt to accelerate its development and maturation

## Conclusion and Perspectives

GC-derived exosomes partially promote occurrence of PM *via* stromal cell remodeling, angiogenesis, immunosuppression, and oncogenic reprogramming. In addition, tumor-reprogrammed stromal cells also secret exosomes absorbed by tumor cells to the malignant phenotype of GC cells. Ever since exosomes have become the new focus of scientific research, significant progress has been made in revealing the contribution of exosomes in conditioning the GC cells for subsequent metastatic processes. Exosomes, acting as correspondents between cells, present substantial effects on shaping the tumor microenvironment, especially pre-metastatic niches. Although exosomes are involved in all steps of PM, more explorations about underlying mechanisms are still lacking. Fortunately, emerging technologies (liquid biopsy and exosomes media) may help early diagnosis of PM by screening exosome miRNAs and exosome-based treatment by transferring anti-tumor drugs and restricting exosomes homing in PM.

## Author Contributions

Conceptualization, LT. Investigation, XC. Writing—original draft preparation, XC. Writing—review and editing, XC, HW, CC, and YH. Supervision, WZ. Project administration, HW. Funding acquisition, LT. All authors contributed to the article and approved the submitted version.

## Funding

This study was supported by the project of the Regional Diagnosis and Treatment Center of the Health Planning Committee (No. JBZX-201903).

## Conflict of Interest

The authors declare that the research was conducted in the absence of any commercial or financial relationships that could be construed as a potential conflict of interest.
